# Dr. Alder's Proofs of His Theory from the Remote Causes of Diseases and Fevers

**Published:** 1810-06

**Authors:** Thomas Alder

**Affiliations:** Edinburgh


### Dr. Alder's *Proofs of his Theory from the remote Causes of Diseases and Fevers.*

( In Continuation. )

DR. HAYGARTH has sharply remarked upon Mr. Webster, that "with a poet's eye with a fine *phrenzy* rolling," he has glanced from earth to heaven, and from heaven to earth, and so forth; a remark, however, which nobody will make upon Dr. Haygarth or his followers; for, (speaking without any hyperbole) when *they* wish to hurl Mr. Webster and his work into irrecoverable contempt, merely for placing before the world *historical truths*, *they* shut their eyes most obstinately and wilfully against all phænomena, whether on the earth, or in the heavens, in the waters or in air, and fix them only on such circumstances as give some countenance to an idea which they had *previously conceived;*—an idea I will not say conceived with the fine phrenzy of a *poet's* imagination, but certainly by no means the offspring of fair induction, but altogether a child of narrow views, fancy, and mere supposition: for shutting their eyes against all circumstances, which we must all know *do* cause fevers, and which, all authors, as well as Mr. Webster, shew *prevail, they* have fixed their mind upon one which they suppose *might* cause them, *(though in this they are mistaken;)* and then, which is nearly equally surprising, they have, *(without ever proving the existence of* THIS) set themselves to work to fancy how it could so extensively ACT; and the result of this, their *second fancy,* is more monstrous than the child of their first, viz. fomites! objects which are ten thousand times more to be dreaded than a fever patient himself! Such monsters of desolation these, *(if we trust their* ACCOUNTS) that no person in the land is, or can be safe, from them; and, indeed, if these accounts about fomites had been true, we must all have been destroyed long ago: for these do every thing they effect after a week, a month, a year; after they have been exposed to the air, the sun, and to water; and yet they are made to kill *one*, when twenty or *forty*, &c. others have been much *more* exposed to them (often *previously)* with impunity! while the fever-body from which these derive all their virulence, *is now found by the same gentleman,* to be quite harmless at the distance of a yard, a foot, *an inch!* nay, quite harmless, in contact to those who are *seasoned to contagion!* (another strange fancyspun spun doctrine!) And also to others. But, indeed, Dr. Haygarth's doctrines are now so much losing their influence with their followers, that the most intelligent, clear-sighted, and respectable of these, begin to declare that contagion *immediately from the human body,* will not act, except a person is *predisposed to fever, or there be a certain bad state of the air.*

I shall pass over then these *fancies* of Dr. Haygarth, to dwell upon Mr. Webster's *plain narrative of facts.*—It requires a little exertion to consider those awful convulsions in nature, which *seem,* on most occasions, to be (some or other of them) connected with epidemics. I say *seem,* but that they *often are so,* must appear to the most sceptical, who at all rely on *history, beyond all doubt,* for they have *too often attended,* to be otherwise than *connected;* and *the chain of their connexion too is, for the most part, plain.*

*Volcanic eruptions, &c.* The interior of the earth must be devoured by fires to a great depth; for it has commonly happened, that many volcanoes have been active about the same time, and some of these situated at the distance of some thousand miles from each other; while the eruptions of fire, and in other places of vapours, only where there has been no volcano and earthquakes, (sometimes shaking whole kingdoms, and several of them, many of these kingdoms at a great distance,) also meteors, droughts, &c. that is, most of the other phænomena happening about the same period so constantly, and at great distances of space, can no more be accounted for than the volcanic eruptions, but by attributing them (as Mr. Webster has for the most part done) to fires often being active within the earth very extensively at the same period. Mr. Webster does not seem to suppose this general activity of *internal fires* depends upon their *communicating,* but to their having some *common cause,* he thinks *electricity;* and he thinks he is confirmed in this latter opinion by the comets and meteors, which at the same time have been frequent. But I rather think their general activity depends much upon their *communication;* and that this is owing to their great *depth:* for fires which evolve gases, so as to shake *whole kingdoms* at once, may well be supposed to be *a thousand miles deep, or much more.* The same earthquake, indeed, will frequently be felt for much more than a thousand miles in extent. The earthquake which destroyed Lisbon, was felt in America. And Mr. Webster's conjecture about the synchronous activity of internal fires being owing to *electricity,* receives no confirmation from meteors being prevalent when internal internal fires are active, because it does not appear that these are *electric matter;* they mostly appear no other than *ignes fatui* on a large scale, and in the higher regions; they do not move *like* electric matter, but like a *slow combustion of hydrogenous or phosphoric matter,* OR BOTH; and they are like the latter too, and not like electric matter, when they are more stationary and permanent; and they have been *seen to arise from volcanoes.*—Besides, we do not find that the *aurora borealis* was noticed among the dreadful phænomena; and we now perceive this *very common,* (though it is supposed to be owing to electricity) without observing *any bad consequences follow it, or any thing bad prevailing with it.* I have noticed it here at Edinburgh most wonderfully striking, (covering with its vivid dances one-eighth of the sky) without perceiving any thing unusual or bad preceding it, prevailing with it, or succeeding it.—And I do not think that the appearance of comets has proved very much for Mr. Webster, though I cannot say that planets' are without magnetic or electric power, as these have but *rarely* been joined with his other appearances; for it is evident he has been *misled by his authors,* and has often mentioned *a comet appeared,* when there could have been *no more than a* METEOR; as he has mentioned *many* more comets than astronomers have any account of. I maintain then that some internal fires are very *deep,* (perhaps as deep as the centre of the earth;) though no doubt others are superficial; and I believe that many of these fires *communicate.*

But these positions being allowed, we have at once a clear theory of all these strange and awful appearances which are so apt to prevail together. For such fires may shake (in different places) the whole earth, by evolving and rarifying gasses: or they may shake only a few kingdoms, or one kingdom. They may cause many volcanoes to be active about the same period, (however distant these may be) or they may vent themselves at a few, at two or one. They may discharge their vapours into the sea, as well as on land, and thus cause the sea to swell, to grow boisterous, and if near shore, to overflow its banks, and they may disease the fish in it to a great extent, or kill them; they may give a sudden or great agitation to the air, change the currents in it, and render it as unwholesome to land animals, as they may the sea to fishes. And they may send out such an abundance of deadly gas, as is sufficient to cause mortality throughout the world, while at the same time, they may rarefy it so, that as soon as it breaks forth, *it may ascend*

cend

*cend to the higher regions,* and so be distributed to distant places, and descend according to circumstances. But if such fires *may do* such things, it is to me pretty clear that they *have done them;* for all these things have often happened, and it does not appear *to me* that they can be attributed to any other cause. And according to the above account, it cannot be surprising that volcanic eruptions, earthquakes, and such like, *precede* or *follow* epidemics, and *are yet* connected with them; but, on the contrary, it would be strange if they often did not; for morbiferous gasses may be, and we know often are, experienced *long before* volcanic explosions, or long *after* them. And such gasses may either spread *immediately* over the surface of the earth, or they may ascend and mix with the clouds, or rise above them, and not descend for *some time.* And many expirations of gasses, which prove lethiferous, may happen before earthquakes, or any sign of agitation in volcanoes. And if we rely on respectable *history,* we may say such things *often do happen.*

I see no more difficulty in supposing that such fires as I am speaking of, may generate and discharge *noxious vapour* sufficient to *disease* the world, than that they can raise and emit enough to *shake it* by earthquakes, which latter, however, it may be *more clear* that they actually do. But I think it is altogether as impossible to attribute many epidemics to any *other* cause, as it is earthquakes, or more so; and I do not believe, at the same time, that that vast abundance of poisonous gas, which comes often for many times a year, and for some days at a time, from Æthiopia, can be, *with any shew of reason,* judged to arise from any other source than volcanoes.

Heat and dryness of the earth, drying up of springs, failure of crops, and famine, evidently *may* arise from internal fires, and when *conjoined,* can be attributed to no other circumstance: and as fires do not burn without generating and emitting *vapour,* these phænomena indicate that the air either is or will be bad *somewhere.* An abundant generation of insects, as well as putrefaction, *may* sometimes, in some measure, be owing to the heat of the earth; great armies of locusts may be owing to their generation having been abundant that season, and their flight to distant countries may arise from their food being scanty at home. A *moist season* naturally makes the air unhealthy. Thick fogs, darkness from vapour; fœtid clouds or dews, speak as plainly *as a moist season,* what the state of the air is. Looming, &c. or a state of the air which makes objects jects seem different from what they are, proves a vapour in the atmosphere; and as this cannot be either wholesome or totally indifferent, *it must be a bad one.* Great disease among beasts, sheep, or cats, &c may, by Contagionists, it is true, be said to be owing to "Contagion;" but disease among fish and the flight of birds cannot; and as these, and all the rest, indicate the prevalence of much noxious vapour; and this actually is able of itself to disease or kill cattle, &c. I do not see why the interference of any unnecessary agent should be supposed, *when none such is proved.* It is *contrary to our laws,* to admit a *supposititious* cause, when we can find a real one is present, and sufficient to produce the effects.

The numerous diseases, whether inflammatory, febrile, apoplectic, or mental, &c. which Mr. Webster has mentioned being epidemic, and evidently from something in the atmosphere, are many of them, such as were never *suspected to spread by* CONTAGION; as pneumonia, the intermitting fever, apoplexy, (and the various tendencies to it) and mania, with some others; indeed, it is now ascertained *the bilious fever* does not; but all of the complaints he noticed are such as I have maintained, *may arise* from an impeded pulmonary transmission, whether this were caused chiefly by bad air, or by bad air equally with pain, or some other agent. And where there is nothing to prevent us, it is better to attribute them all to similar causes, when these causes *are known to be applied, and can produce them.* But there is *nothing to prevent us; for the existence of* CONTAGION *was not proved in a single case,* and in many of the cases it was positively said *not* to be present. If we were to allow contagion to act too, as well as bad air, &c. we should therefore allow what, in some cases, it was proved *was not present,* and we should in the rest admit an *unnecessary* as well as a *supposititious cause.* The small-pox and measles greatly differ in this point, for two reasons;—*first, they could not be caused without contagion; and secondly, contagion was proved to exist with them.* These complaints are totally different in their nature from any of the others; very different from fevers, though they certainly come nearer to these than to the rest. There evidently is a complete bar between them, by the one occurring only once during life, and by the other happening as often as the causes of it are applied in sufficient violence. But small-pox and the measles have often been more epidemic and mortal, under a bad state of the air, than at other times. The bad state of the air, indeed, clearly accounts for their being more mortal where they attack; and it also furnishes us with some reason for explaining the frequency of these complaints under it, for we are certain, that from the violence or malignity of these diseases at that time, *much more contagion must be generated* by each, (I suppose ten or twenty times more) *than when the air is good;*—perhaps *a hundred times more;* and it is not improbable, that the state of the air rendering the body liable to disease, or actually *unwell,* causes it to be infected by contagion more readily.

Almost all that which has preceded, tends to prove the prevalence and abundance of bad air, from *one source alone,* viz. terrestrial expirations; and this appears to have been by far the most copious source. It is true, we have read of moist or wet seasons, of thick fogs, dark or fœtid clouds, rotten dews, &c. accompanying epidemics; and of these latter being more frequent and fatal in moist or marshy situations, in badly-ventilated places, and near the slaughter-houses at Cork, &c.: and *most* of these things shew a bad air, *not derived from the interior of the earth,* to have done much mischief: but the evil done by them does not appear nearly so extensive *as that occasioned by bad air, derived from the interior of the earth;* and indeed, when descended clouds prove very morbiferous, we have reason to think they contain some volcanic expirations; for independent of what I have before said, it is clear these do mix with the clouds from the sooty snow which fell (after an eruption of Mount Heckla) in Scotland.

Disease among beasts and sheep may seem to indicate bad air that arises from the surface of the ground; just as diseases among birds or fish, or the flight of birds, (for they generally forsake a place, if they can, when the badness of the air reaches so high as to affect them) appear to prove the bad air then prevailing, comes from another source. But cattle in Sicily and Italy have sometimes died in great abundance, in a great measure, from heavy *volcanic* vapours, (as noticed by Mercatorius, and one or two other authors.) It has sometimes happened, that the vapours from Heckla as well as Ætna and Vesuvius, have been confined to a small district around their sources, producing endemics there in the human species as much as in cattle; but when we consider the weight of the sulphureous gas, and the carbonic acid, and consider that the latter from some caverns is discharged nearly pure, and that the former has evidently been (from the sulphureous smell) sometimes copiously emitted from volcanos, we shall not be surprised, surprised, if, in volcanic countries, terrestrial expirations often affected cattle more than men; though we may, *at the same time*, admit, that the diseases in cattle are *in general* owing most to vapours which arise from the surface of the earth, or are *precipitated* from the higher parts of the atmosphere. —— I come now to notice those *tides of the air, evaporation, dews, thaws, the descent of clouds, &c.* Every body is subject to gravitating attraction (cæteris paribus) in proportion to its weight; and every fluid body is moved in proportion to its weight and mobility. The atmosphere being so much more mobile than water, must be much more moved by planetary attraction. It will have greater tides than the sea. These might be of little consequence in my present undertaking, did not the planets, in making these tides, draw all the vapours, as well as pure air, and also facilitate evaporation. They must do so according to all known laws:—*but we find they do.* Every body looks to the full and new moon for a change of weather; and long observation has established the fact, that the air, at such times, is much more apt to be very cloudy. But the clouds drawn together at such times, are sometimes heavy, and roll upon, or lie on the ground, or fall in rain, and sometimes they are light, and ascend so high, that the earth and its inhabitants are nearly free from them. This is a theory which the best established of the laws of Nature, and common and long observation, both confirm.

But taking this for granted, we have a solution of some *more difficulties;* for the greater prevalency and mortality of diseases at *these periods*, is a fact established beyond all cause for doubt. Dr. Johnson has, it is true, thought fit to assert, the weather has no influence upon the spirits and the mind;* and Dr. Heberden has not allowed that lunacy (as it is called) is at all influenced by the planets. But great men expect to be indulged in great *fancies.* It is almost impossible for five medical men in a hundred to deny either: though it would be very fair for them to say, that they do not invariable *hold.* They may have seen people not perceptibly affected by the weather; and they may have seen numerous examples, where lunacy was not influenced by the moon. I have known a person, who said he always felt most pleasant in a moist atmosphere; and cheerfulness, a good state of the alimentary canal and pulmonic system, with a dry house, may prevent it from having much influence upon most: while, on the other side, a foggy, or wet atmosphere, in these lower regions, is not,

 as as I have said, by any means a constant attendant upon planetary attraction; the vapours drawn together may be greatly rarified and elevated, or they may have quickly fallen in showers, so as to have purified the air. On some occasions too, as in frosty weather, there may have been no vapours to be attracted. But fevers are the diseases which I have now to consider. Mr. Balfour has, from long observation affirmed, that those in India have often been much more fatal and frequent at the full and new moon than at other times; and his observations are confirmed by the public testimony of a great number of military gentlemen, who resided there when he did. A learned gentleman thinks he gets over this by saying people would be tempted to walk by moon-light, and in walking, would expose themselves to noxious dews; but this, at any rate, (supposing it was a circumstance overlooked) does not account for aggravations of fever, except (which is admitting my theory) that the vapours are greater at these times; nor does it explain the attacks at the new moon. Dr. Lind speaks in confirmation of the fact too, that fevers are more apt to attack, and to recur, at the full and new moon. But Dr. Jackson, that the planetary influence in the West Indies, and in the United States of America, *where he has* resided, is pernicious between the new and full moon; and this, (though brought to support the opinion) by appearing so at variance with the preceding observations, has a great tendency to destroy it altogether. But Mr. Guthrie observes, "when the sun becomes vertical, or nearly so, in the West Indies, he draws after him such a vast body of clouds, as shield the people below from his direct beams, and then uniting and falling in rain, cool the air and refresh the Country;" and it is most probable, that the moon may often have a somewhat similar effect; though, I apprehend, the vapour, which, in such cases, keeps under the sun, is owing, in a slight degree, to some other causes than his attraction; that is, to his heat rarifying the air underneath, and making room for vapours; and to the rotatory motion of the earth bringing up vapours on one side faster than the circumstances above named will let them depart on the other. But as the sun's rays are known to heat bodies immediately which reflect them, and those which freely transmit them, *only by reflexion*, the vapour drawn together is likely to be heated and rarified very much near the earth, and to become condensed at a height much more above the reach of animal mal respiration than the earth's reflexions. And this seems another reason why the vapours the sun brings underneath him often do more good than harm.

I am, &c.

THOMAS ALDER.

*Edinburgh, May* 1, 1810.

( To be continued. )

